# Unfolding Studies of the Cysteine Protease Baupain, a Papain-Like Enzyme from Leaves of *Bauhinia forficata*: Effect of pH, Guanidine Hydrochloride and Temperature

**DOI:** 10.3390/molecules19010233

**Published:** 2013-12-24

**Authors:** Rosemeire A Silva-Lucca, Sheila S Andrade, Rodrigo Silva Ferreira, Misako U. Sampaio, Maria Luiza V. Oliva

**Affiliations:** 1 Departamento de Bioquímica, Universidade Federal de São Paulo, Rua Três de Maio, 100, São Paulo 04044-020, SP, Brazil; E-Mails: roselucca@uol.com.br (R.A.S.-L.); sheilasa@gmail.com (S.S.A.); rodrigobioq@gmail.com (R.S.F.); misako.bioq@epm.br (M.U.S.); 2 Centro de Engenharias e Ciências Exatas, Universidade Estadual do Oeste do Paraná, Rua da Faculdade, 645, Toledo 85903-000, PR, Brazil

**Keywords:** baupain, circular dichroism, cysteine protease, papain, protein conformation

## Abstract

Baupain belongs to the α+β class of proteins with a secondary structure-content of 44% α-helix, 16% β-sheet and 12% β-turn. The structural transition induced by pH was found to be noncooperative, with no important differences observed in the pH range from 3.0 to 10.5. At pH 2.0 the protein presented substantial non-native structure with strong ANS binding. Guanidine hydrochloride (GdnHCl)-induced unfolding did not change the protein structure significantly until 4.0 M, indicating the high rigidity of the molecule. The unfolding was cooperative, as seen by the sigmoidal transition curves with midpoints at 4.7 ± 0.2 M and 5.0 ± 0.2 M GdnHCl, as measured by CD and fluorescence spectroscopy. A red shift of 7 nm in intrinsic fluorescence was observed with 6.0 M GdnHCl. Temperature-induced unfolding of baupain was incomplete, and at least 35% of the native structure of the protein was retained, even at high temperature (90 °C). Baupain showed characteristics of a molten globule state, due to preferential ANS binding at pH 2.0 in comparison to the native form (pH 7.0) and completely unfolded (6.0 M GdnHCl) state. Combined with information about N-terminal sequence similarity, these results allow us to include baupain in the papain superfamily.

## 1. Introduction

We have previously reported the biochemical characterization of a cysteine protease from *Bauhinia forficata* leaves, named baupain [1]. *Bauhinia forficata* leaves extract is used in folk therapy in Brazil as an infusion for diabetes mellitus. The results of baupain characterization showed that it is a 23,000 Da single polypeptide chain protein, active at neutral pH, and belonging to the CA clan of the papain family. Our findings also showed that baupain has kininogenase activity [1] and blocks thrombin-induced human platelet aggregation, possibly by unspecific cleavage of protease activated receptors 1 (PAR1) [2].

The folded conformation adopted by a protein is generally governed by its amino acid sequence, suggesting an underlying stereochemical code for protein folding. Unfolding/refolding of many globular proteins has been well described in terms of a two-state (native↔denatured) model [3]. However, how a protein that is synthesized as a linear unfolded polypeptide form rapidly folds into its biologically active structure, even though there are a large number of potential conformational states, still remains an intriguing and unsolved question. Indeed, characterization of protein folding and the role played by intermediate states in this process is important, since it can provide insights into how mutated proteins cause genetic diseases, biotechnological problems and delayed drug design development [4].

The molten globule state, a non-native compact state of a protein, is typically regarded as a general intermediate in protein folding. This state is characterized by the presence of significant amount of secondary structure, lack of most specific tertiary structures associated with tight packing of side chains, and the presence of a loosely packed hydrophobic core, which is accessible to solvent molecules. Molten globule structures have been observed for many proteins under different conditions such as low and high pH or in low concentration of the denaturants urea and guanidine hydrochloride (GdnHCl) [5]. Papain exhibits the characteristics of a molten globule state at acidic conditions, as it contains a substantial amount of secondary structure and significantly disordered tertiary structure with exposed hydrophobic regions [6,7]. Similar folding behavior has also been observed with other plant cysteine proteases such as stem bromelain [8] and ficin [9].

Protein unfolding induced by chemical denaturants, pH or temperature are common approaches to study proteins *in vitro*. Here, we investigated the conformational behavior of baupain in solution as a function of pH, temperature and chemical denaturants. The results revealed evidence for a molten globule-like state at acidic conditions. The influence of temperature on enzyme proteolytic activity on synthetic substrates was also investigated. Additional studies about the conformational behavior of similar proteases from different members of the cysteine protease family would be important towards elucidating the folding pathway of this class of proteins.

## 2. Results and Discussion

### 2.1. Circular Dichroism of Native Baupain and Secondary Structure Estimation

**Figure 1:**
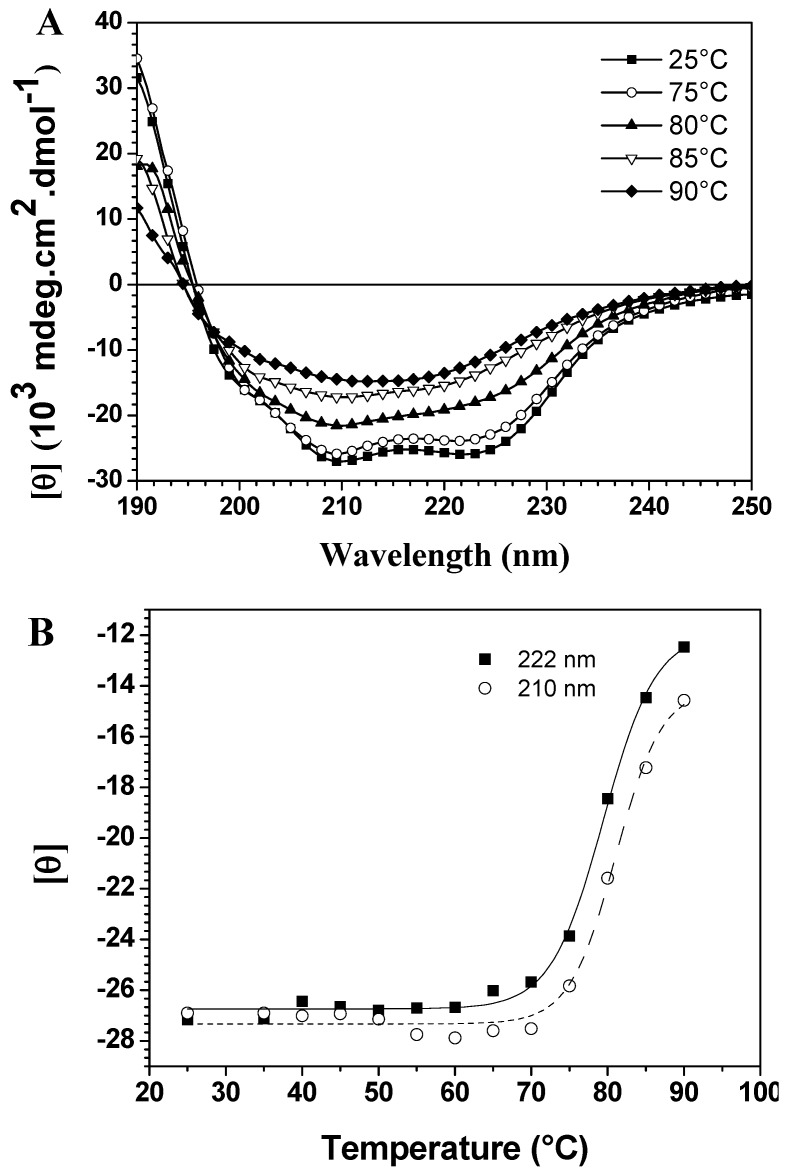
Temperature dependent conformational changes of baupain in 10 mM phosphate/borate/acetate (PBA), pH 7.0. (**A**) Far UV CD spectra of baupain (0.23 μM) at different temperatures. (**B**) Relative changes of mean residual ellipticity values (MRE) at 210 nm (**squares**) and 222 nm (**circles**) as a function of temperature. The sigmoidal fit of these points provides a midpoint around 80 °C for this transition.

Circular dichroism and fluorescence spectroscopy of proteins are widely used to monitor conformational changes of proteins with changes in solvent composition. The CD spectrum of baupain in 10 mM PBA solution at pH 7.0 (Figure 1A, squares) showed two minima, one at 222 nm, related to the strong hydrogen-bonding environment of α-helices, and another at 210 nm. This is similar to the papain spectrum that shows two bands at 222 and 208 nm, characteristic of an α-helix structure [10]. Analysis of secondary structure content using the CDPro software package with the SELCON3, CONTINLL, CDSSTR programs yielded 44% α-helix, 16% β-sheet, 12% β-turn, and 28% unordered structures, with a standard deviation of 2% [1]. Cluster analysis showed that baupain belongs to the α+β tertiary structure class, like other cysteine proteases, for example procerain and bromelain [8,11]. This classification was corroborated by the results of CDPro estimation (44% α-helix and 16% β-sheet) and spectrum sharpness (with the band at 210 nm slightly more intense than that at 222 nm), which is similar to the papain spectrum that shows two bands (208 and 222 nm) in neutral pH [12]. Together with biochemical data obtained previously [1], the current results allow us to classify baupain in the clan CA of the papain family.

### 2.2. Thermal Denaturation

CD spectroscopy in the far-UV region can monitor conformational changes in the polypeptide backbone. Thermal assays were carried out in the 25 °C to 90 °C range in 5 °C intervals. The spectra in Figure 1A show thermal scans at different temperatures, demonstrating significant differences in thermal stability due to the loss of two typical bands with increased temperature. On the other hand, temperature-induced unfolding of baupain was incomplete since the spectrum at 90 °C still displays a band centered at 215 nm, characteristic of a β-sheet structure, although less intense. To better analyze these conformational changes, the mean residual ellipticities [θ] at 210 nm and 222 nm (MRE_210_ and MRE_222_) were plotted against temperature, as shown in Figure 1B. In this case, baupain reveals pronounced structural stability until 70 °C, with only slight fluctuations in the MRE values. A sigmoidal fit to these points yielded a midpoint around 80 °C. Corroborating the spectral changes, a significant variation in secondary structure content occurs in the transition temperature from 80 to 85 °C. Namely, at 80 baupain showed 41% α-helix, 16% β-sheet, 19% β-turn and 26% unordered structure content, whereas at 85 °C the α-helix content decreased to 24% and the β-sheet and unordered structure contents increased to 24% and 33%, respectively. These last values were maintained at 90 °C. In addition, the data show high protein structural stability, possibly assigned to β-sheet structures since they are more resistant and less susceptible to unfolding [4]. The transition was irreversible, since a refolding attempt by decreasing the temperature under the same unfolding conditions, did not lead to recovery of the original structural characteristics (data not shown).

**Figure 2:**
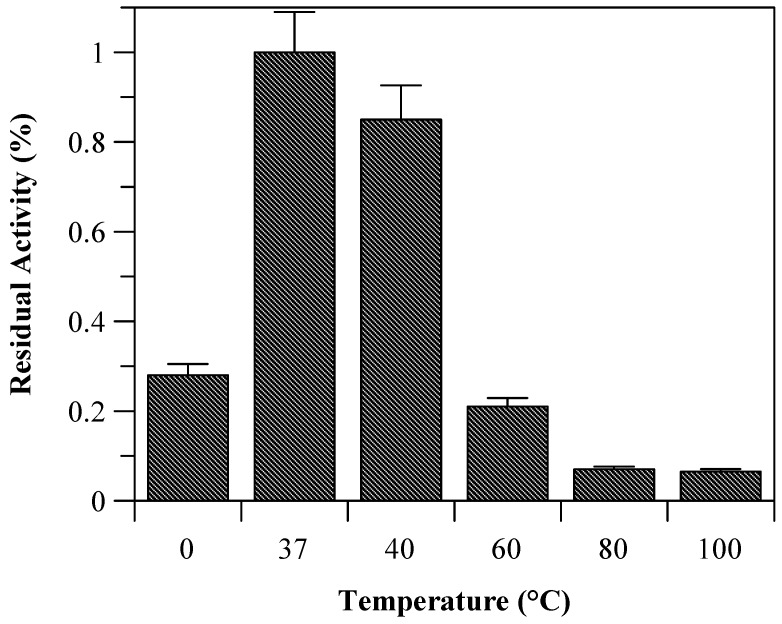
Residual activity of baupain (0.050 mg/mL) towards Z-Phe-Arg-MCA (0.4 mM) determined at different temperature.

Due to the incomplete thermal unfolding, baupain activity was investigated with regard to its hydrolytic activity on the Z-Phe-Arg-MCA substrate (Figure 2). Baupain maximum activity was achieved at 37 and 40 °C, and very low activity was observed at extreme temperatures (60, 80, and 100 °C), similar to the behavior observed with papain-like cysteine proteinases [10]. The data show that although the secondary structure was maintained until 70 °C (Figure 1), baupain was unable to cleave the substrate at this temperature. These results suggest that changes in tertiary structure led to a modification of the enzyme active site, thus affecting baupain function.

### 2.3. Guanidine Hydrochloride Unfolding

Chaotropic agents such as GdnHCl or urea are commonly used in unfolding/refolding studies, although the unfolding mechanism is not clear. These studies were followed by far-CD, fluorescence and ANS binding assays.

**Figure 3:**
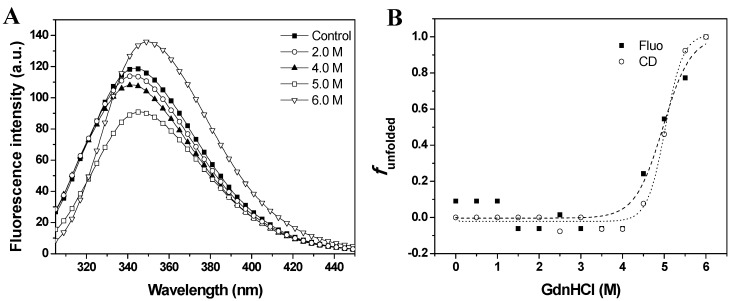
GdnHCl-induced unfolding of baupain in 10 mM phosphate/borate/acetate (PBA), pH 7.0, 25 °C, as a function of GdnHCl concentration. (**A**) Intrinsic fluorescence emission spectra were measured in the absence and in the presence of chaotropic agent. (**B**) Normalized transition curves for GdnHCl-induced transition. The unfolded fractions were calculated with equation 1 using the fluorescence emission maxima (λ_max_, **squares**) and mean residual ellipticity values at 222 nm (**circles**). The sigmoidal fits for CD (·····) and fluorescence data (----) defined the midpoints at 4.7 ± 0.2 M and 5.0 ± 0.2 M GdnHCl.

Intrinsic fluorescence emission provides a sensitive probe to characterize proteins by monitoring tryptophan residues that are very sensitive to the polarity of the environment. The wavelength at the emission maximum (λ_max_) and the fluorescence intensity can be used to characterize proteins and to determine conformational changes [13]. The effects of GdnHCl on fluorescence emission spectra are shown in Figure 3A. The intrinsic fluorescence emission of baupain at pH 7.0 displayed an emission maximum (λ_max_) at 343 nm, suggesting that tryptophan residues were at least partially exposed to solvent. The fluorescence emission spectra of baupain showed no significant change in intensity or in the emission maxima in 0–3.5 M GdnHCl. However, the fluorescence intensity increased 13% in 6.0 M GdnHCl, along with a red-shift of 7 nm in emission maximum (from 343 to 350 nm), indicating exposure of tryptophan to a more polar environment.

Gradual loss of secondary structure of the enzyme is demonstrated by a marked decrease in the typical CD band at 222 nm (from 24,582 deg·cm^2^·dmol^−1^ to 4,555 deg·cm^2^·dmol^−1^) in 6.0 M GdnHCl (data not shown). To better examine the conformational changes induced by GdnHCl on baupain at pH 7.0, the unfolded fractions were calculated by Equation (1) (see Experimental, Section 3.6) and plotted against GdnHCl concentration values, using as signals the mean residue ellipticity intensities at 222 nm (α_obs_, circles) and fluorescence emission maxima (λ_max_, squares) (Figure 3B). The sigmoidal curves suggest the presence of cooperative conformational changes induced by GdnHCl unfolding in a single step from native to unfolded form (N→U), with no detectable intermediates. From the sigmoidal fits we determined the midpoints 4.7 ± 0.2 M (from CD data) and 5.0 ± 0.2 M GdnHCl (from fluorescence data). In order to analyze global conformation changes induced by GdnHCl, the exposure of the hydrophobic surfaces of baupain was monitored by changes in the fluorescence of the ANS dye. This probe has low fluorescence intensity in the presence of a hydrophilic environment, whereas ANS binding to hydrophobic regions leads to a blue-shift of the emission maximum and a pronounced increased in fluorescence intensity. ANS (23 µM) was added to baupain (0.23 µM) in the absence and presence of different concentrations of GdnHCl at pH 7.0*.* We noted no significant increase in ANS fluorescence that would indicate the presence of hydrated hydrophobic surfaces for ANS binding (data not shown), as also described by Khurana *et al.* [14].

### 2.4. Acid-Induced Unfolding

**Figure 4:**
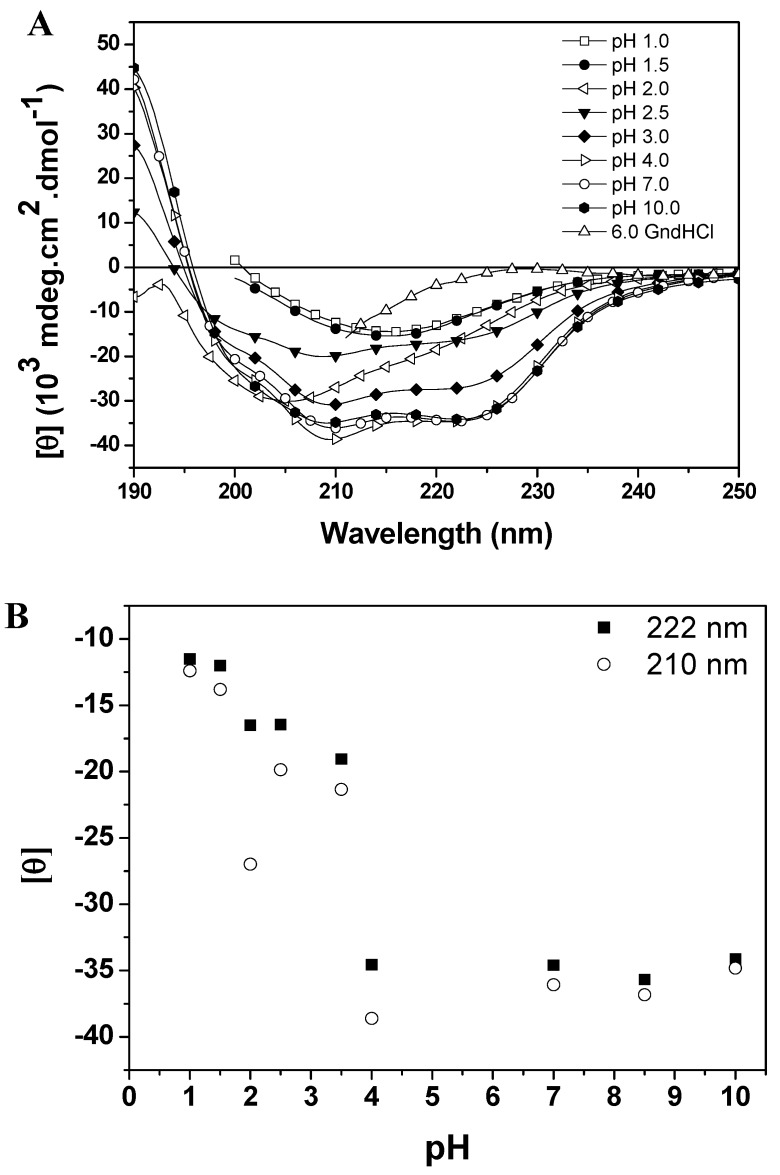
pH dependent conformational changes of baupain at 25 °C. (**A**) Far UV CD spectra of baupain (0.23 μM) under different pHs. (**B**) Relative changes of mean residual ellipticity values (MRE) at 210 nm (**circles**) and 222 nm (**squares**) as a function of pH. For comparison, the CD spectrum of baupain in 6.0 M GdnHCl is also shown.

An important factor in protein conformational stability is the pH of the environment, which can change the net charge of biomolecules, and thus many proteins denature at extremes pH due to the presence of repulsive forces [14,15]. However, the acid denaturation of proteins often results in unfolded states with less denaturation than that obtained by high concentrations of chaotropic agents such as GdnHCl or urea [8,9,10]. This incomplete denaturation may be due to electrostatic repulsion forces that are insufficient to overtake the interactions that favor folding, such as hydrophobic interactions, salt bridges and disulfide bonds. Baupain conformational changes induced by pH variation were monitored by far-CD, fluorescence and ANS binding studies. The effect of pH on the secondary structure of the protein is shown in Figure 4A (CD spectra of baupain as a function of pH) and 4B (mean residue ellipticity, MRE, *versus* pH). The MRE_210_ and MRE_222_ values for baupain remained unchanged in the pH range 4.0–10.5 (Figure 4B), with the protein retaining most of its α-helices and β structures (44% and 16% at pH 7.0, and 40% and 15% at pH 4.0). For pH values below 3.5 there was a gradual decrease of the characteristic bands of CD (at 210 and 222 nm) with the emergence of a low intensity band at 215 nm at pHs 1.0 and 1.5 (Figure 4A). On the other hand, at pH 2.0 the protein retained a significant amount of secondary structure as shown by the value of MRE_210_ (28,000 deg·cm^2^·dmol^−1^). Finally, below the pH 2 most of the secondary structure was lost, with a pronounced decrease of MRE values and a partial unfolding of the enzyme. These results have been observed for other cysteine proteases and may suggest the presence of an intermediate state (molten globule) at pH 2.0. In fact, a weak cooperative process is observed in Figure 4B, suggesting an intermediate state. Baupain also maintains its secondary structure in a wide pH range, which is a peculiar feature also characteristic of other papain-like enzymes [9,11]. Thus, the pH-secondary conformational stability of baupain was further studied by evaluating the effect of pH on tertiary structure changes.

**Figure 5:**
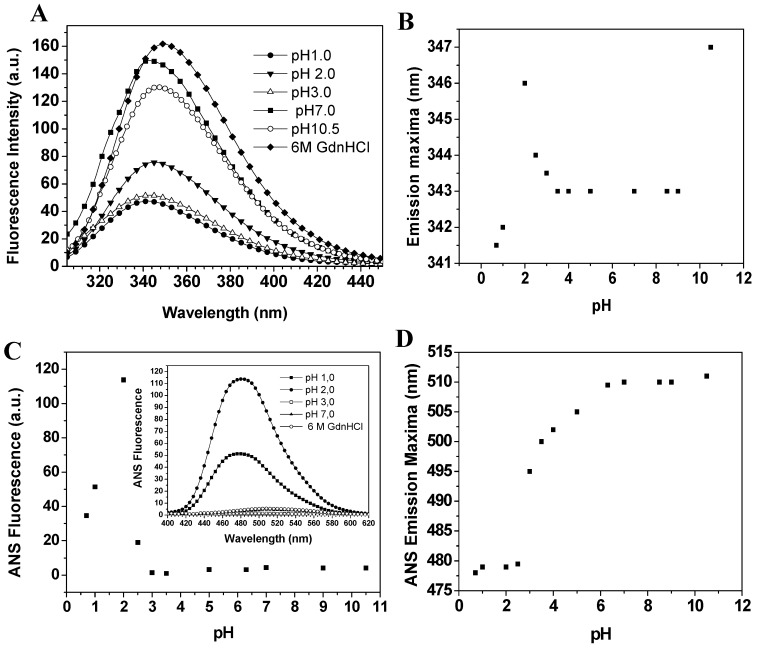
Effect of pH on the fluorescence of baupain (0.23 μM) at 25 °C. (**A**) Intrinsic fluorescence emission spectra and (**B**) emission maxima (λ_max_,) plotted against pH values. (**C**) ANS fluorescence intensity as a function of pH and ANS fluorescence emission spectra of baupain at pH 2.0, pH 7.0 and GdnHCL 6.0 M (insert). (**D**) pH dependence of ANS emission maxima (λ_max_) for baupain.

Baupain fluorescence emission shows maximum intensity at pH 7.0, 50% intensity at pH 2.0, and around 67% at pHs 1.0 and 3.0. At basic pH, the fluorescence intensity decreases to 13% (Figure 5A). It is worth pointing out that the emission maximum (λ_max_) was unchanged in the pH range 3.0–10.0 (Figure 5B). However, at pH 2.0 a slight red shift from 343 to 346 nm was observed for the emission maximum, suggesting that tryptophan residues are exposed to a slightly more hydrophobic environment, which would be compatible with a more unordered structure. Furthermore, the CD spectra still show a significant content of secondary structure at pH 2.0. The effect of pH in the tertiary structure of baupain was also monitored using ANS dye.

ANS fluorescence intensity changed with pH, reaching a maximum at pH 2.0 (28-fold increase, Figure 5C). Below pH 2.0, ANS fluorescence intensity was twelve times larger than at pH 7.0, suggesting the structural reorganization of the enzyme to protect its hydrophobic regions from the solvent. The emission maximum shifted to shorter wavelength (478 nm) at pH 2.0, in comparison to the spectrum at pH 7.0 (510 nm, Figure 5D). This blue shift in emission maximum together with the large increase of fluorescence intensity indicates an extensive solvent exposure of non-polar clusters. Taken together, the CD spectra (Figure 4A), intrinsic fluorescence spectra (Figure 5A) and ANS fluorescence spectra (Figure 5C, insert) at pHs 2.0 and 7.0 and in 6.0 M GdnHCl reveal that the state of baupain molecules under acidic conditions is different from that under neutral pH or relative to the unfolded state.

## 3. Experimental

### 3.1. Isolation of Enzyme

**Figure 6:**
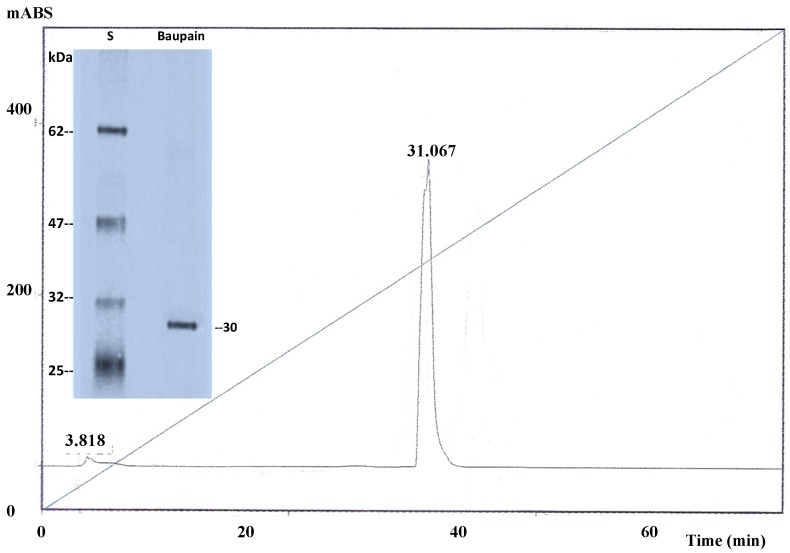
HPLC analyses of the cysteine protease baupain. Baupain (10 µg) from Con A-Sepharose was analyzed using a binary HPLC system from Shimadzu with a SPD-10AV UV-visible light detector and a RF-10AXL fluorescence detector (Shimadzu Corporation, Tokyo, Japan) coupled with an Ultrasphere C-18 column (5 mm, 4.6 × 150 mm). Elution was performed with solvent systems A (TFA/H_2_O; 1:1000) and B (TFA/ACN/H_2_O; 1:900:100) at a flow rate of 1 mL/min and a 5%–100% gradient of B over 60 min. The HPLC column eluates were monitored for their absorbance at 215 nm. Inset: SDS-PAGE (12%) of the cysteine protease baupain from *Bauhinia forficata* leaves. Baupain (10 µg) from Con A-Sepharose under reducing conditions, Coomassie blue staining; (**S**), standard proteins, β-lactoglobulin A (25 kDa), triosephosphate isomerase (32 kDA), aldolase (47 kDa), glutamic dehydrogenase (62 kDa).

Baupain was purified as described by Andrade *et al.* [1]. Briefly, the proteins were precipated with acetone from crude extract (7%) and applied on a size exclusion Sephadex G-25 column followed by canecystatin-Sepharose and Con A Sepharose chromatographies. The fractions containing enzyme activity activity (SDS-PAGE, Figure 6, inset) were purified by HPLC on a μ-Bondapak C18 reverse phase column (Figure 6) for subsequent automated Edman degradation, which allowed identification of the N-terminal sequence IPEYVDWRQQ. Baupain shows similarity to members of the CA family of cysteine proteases, being 90% and 40% identical to papain and bromelain, respectively. Protein concentration was estimated spectrophotometrically at 280 nm as well as by Bradford [16] assay using bovine serum albumin as standard.

### 3.2. Circular Dichroism (CD) Measurements and Secondary Structure Estimative

CD spectra were recorded using a Jasco J-810 spectropolarimeter (Jasco Instruments, Tokyo, Japan) equipped with Peltier thermostatting cuvette holder, over the range of 195–250 nm (far-UV) under constant N_2_ purging. CD spectra of native baupain (0.23 μM) were measured in 10 mM phosphate/borate/acetate buffer (PBA) pH 6.3 and pH 7.0, in a quartz cuvette of 0.1 cm of path-length, at 25 °C. The results were expressed as the mean residue ellipticity, [*θ*], defined as [*θ*] = *θ*_obs_/(10.*C*.*l*.*n*), where *θ*_obs_ is the CD in millidegrees, *C* is the protein concentration (*M*), *l* is the path length of the cuvette (cm), and *n* is the number of amino acid residues (261). The CDPro software was used to estimate the fractions of secondary structures [17] and the Cluster program was used to determine the tertiary structure class of baupain [18].

### 3.3. Intrinsic Fluorescence Measurements and ANS Binding Assays

Steady state fluorescence measurements were carried out on a Hitachi F2500 spectrofluorimeter (Tokyo, Japan) in a 1 cm path length rectangular quartz cuvette. The temperature of the samples was kept at 25 °C using a circulating water bath (Micronal B15, São Paulo, Brazil). Intrinsic fluorescence emission of native baupain (0.23 μM) was measured in 10 mM PBA, at pHs 6.3 and 7.0, with excitation at 295 nm and emission recorded in the range of 305–420 nm. Baseline corrections were carried out with buffer without protein in all cases. In the ANS binding assay, the dye was added to the same samples used for intrinsic fluorescence (23 μM probe and 0.23 μM protein), and incubated for 30 min in the dark. Excitation was carried out at 380 nm and the emission recorded from 400 to 620 nm. Baseline corrections were carried out with ANS solutions in each particular concentration, without protein.

### 3.4. Acid Denaturation of Baupain

Acid denaturation of baupain (0.23 μM) was examined by keeping the enzyme for 15 min in pH ranges of 1.0–2.0 (in a 5 mM KCl-HCl solution), 2.2–3.5 (in a 5 mM glycine buffer), and 3.5–10.5 (in 10 mM PBA). Following far-UV CD measurements, the same samples were examined in a fluorimeter for detection of tryptophan surface exposure in the tertiary structure of baupain, by means of hydrophobic surface analysis with the ANS probe. The measurement conditions were the same described for the native baupain.

### 3.5. Temperature and GuHCl Induced Unfolding

Thermal denaturation measurements for baupain (0.23 μM) at pH 7.0 (native-form) were monitored by far-UV CD spectroscopy, using a Peltier apparatus. The measurements were carried out in the range 25–90 °C, with a scanning rate of 2 °C/min and at 5 °C intervals. Protein sample was incubated for 15 min before measurements. Baupain unfolding at pH 7.0 (native form) and pH 2.0 (acid form) induced by guanidine (GdnHCl) was monitored by far-UV CD and fluorescence spectroscopy. The protein samples (0.23 μM) were incubated in various concentrations of GdnHCl (0.5–6.0 M) for 24 h prior to the analysis. The measurement conditions for CD and fluorescence spectroscopy were the same described for the native baupain.

### 3.6. Analysis of Unfolding Experiments

The unfolding transition curves for baupain, measured by circular dichroism and fluorescence spectroscopy, were analyzed by expressing the data in terms of unfolded fraction (*f*_U_) of baupain, calculated from the equation:

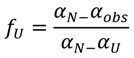
(1)
where α_obs_ is the observed value of the signal at a given denaturant concentration, and α_N_ and α_U_ are the values of native and unfolded protein, respectively. For the CD experiments, the intensity of the bands at 222 and 210 nm were monitored for the native and unfolded states, whereas for the fluorescence measurements the wavelength emission maxima in the fluorescence (λ_max_) spectra were followed.

### 3.7. Enzyme Activity

Proteinase activity was measured using Z–Phe–Arg–MCA (0.4 mM) (Calbiochem Ltda, Darmstadt, Germany) as substrate. Baupain (0.23 μM) was incubated at 37 °C in a microtiter plate in 250 µL final volume of assay buffer [0.1 M sodium phosphate buffer, pH 6.3 containing 0.4 M NaCl, 10 mM EDTA, and 2.0 mM DTT (dithiothreitol)]. The reaction was followed for 10–30 min and stopped by the addition of 50 µL acetic acid 30% (v/v). The fluorescence release was measured on a FluoroCount Packard^TM^ spectrofluorometer (Meriden, CT, USA) set at 355 nm for excitation and 460 nm for emission.

### 3.8. Effect of Temperature on the Enzyme Activity

Prior to the addition of the substrate Z–Phe–Arg–MCA (0.4 mM), 40 µL of baupain (0.23 µM) was pre-incubated at a series of temperatures of 0, 37, 40, 60, 80, 100 °C for 30 min with 100 µL of the 0.1 M sodium phosphate buffer, pH 6.3 containing 0.4 M NaCl, 10 mM EDTA in a final volume of 250 µL by adding 90 µL of distillated water.

## 4. Conclusions

**Figure 7:**
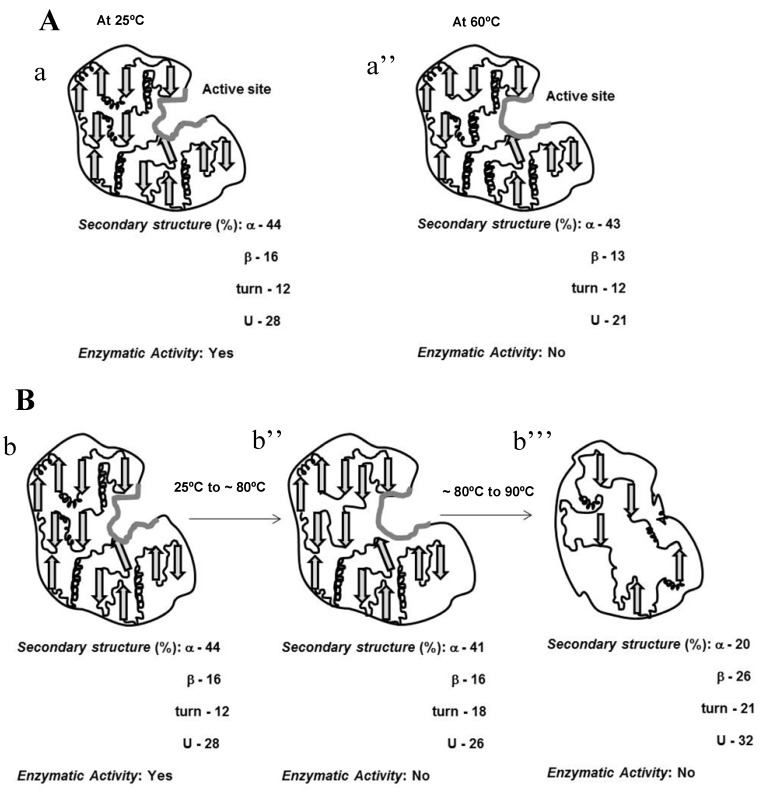
Schematic representation of temperature dependent conformational changes in baupain. (**A**) Conformational changes in the active site (gray line) at 25 °C (a’) and 60 °C (a’’). (**B**) Effect of temperature on the secondary structure of native (b’), at 80 °C (b’’) and 90 °C (b’’’).

In this study we found that baupain, a papain-like enzyme that hinders thrombin-induced human platelet aggregation and shows kinogenase activity [1,2], fully active at 37 °C, displayed incomplete temperature-induced unfolding, where at least 35% of the native structure of the protein was retained even at a high temperature (90 °C), as illustrated in the scheme of Figure 7.

In addition, at 60 °C baupain was unable to cleave the substrate suggesting that changes in tertiary structure lead to a modification of the enzyme active site, thus affecting baupain activity. Exposure of baupain to GdnHCl led to a single step unfolding process (*i.e.*, with no detectable intermediates) from native to unfolded form, with a red shift of 7 nm of intrinsic fluorescence in 6.0 M GdnHCl. Finally, a weak cooperative unfolding was observed when varying the pH. At pH 2.0, baupain still retained a significant content of secondary structure, but without its tertiary packaging, as indicated by ANS binding assays reflecting the exposure of its hydrophobic regions. These results at pH 2.0 demonstrate the presence of a transient intermediate state during unfolding of baupain, possibly a molten globule state, similar to papain and ficin [8,9]. The stability of the secondary structure of baupain in a wide pH range is a peculiar feature of this enzyme, but which is characteristic of others papain-like enzymes [9,11].These findings can help understand the structure–function relationship of proteins (mainly enzymes) under different solvent conditions, and can provide insight into the molecular basis of their structural stability.

## Abbreviations

CD: circular dichroism
ANS: 1-anilino-8-naphthalene sulfonate
GdnHCl: guanidine hydrochloride
PBA: phosphate/borate/acetate buffer
